# A Slight Mistake

**Published:** 1894-07

**Authors:** 


A SLIGHT MISTAKE.—There is a joke being told at the expense of a modest young newspaper man, which is so good it ought to be true, says the *Bristol News*. The young man was recently invited to a party at a residence where the home had lately been blessed with an addition to the family. Accompanied by his best girl he met their kind hostess at the door, and after the customary salutations, asked after the welfare of the baby. The lady was suffering from a severe cold, which made her slightly deaf, and she mistakenly supposed the young man was inquiring about her cold. She replied that though she usually had one every winter, this was the worst one she ever had; it kept her awake at night a good deal at first, and confined her to her bed. Then noticing that the scribe was becoming pale and nervous, she said that she could see by his looks that he was going to have one just like hers, and asked him if he wished to lie down. He has given up inquiring about babies.

